# Improved Functional Outcome After Peripheral Nerve Stimulation of the Impaired Forelimb Post-stroke

**DOI:** 10.3389/fneur.2021.610434

**Published:** 2021-04-20

**Authors:** Shih-Yen Tsai, Jennifer A. Schreiber, Natalie S. Adamczyk, Joanna Y. Wu, Son T. Ton, Ryan C. Hofler, James S. Walter, Timothy E. O'Brien, Gwendolyn L. Kartje, Russ P. Nockels

**Affiliations:** ^1^Edward Hines Jr. Veteran Affairs Hospital, Hines, IL, United States; ^2^Department of Neurological Surgery, Loyola University Medical Center, Maywood, IL, United States; ^3^Department of Mathematics and Statistics and Institute of Environmental Sustainability, Loyola University Chicago, Chicago, IL, United States; ^4^Department of Molecular Pharmacology and Neuroscience, Loyola University Chicago Health Science Division, Chicago, IL, United States

**Keywords:** stroke, recovery, plasticity, middle cerebral artery occlusion, electrical stimulation, implantable device

## Abstract

Lack of blood flow to the brain, i.e., ischemic stroke, results in loss of nerve cells and therefore loss of function in the effected brain regions. There is no effective treatment to improve lost function except restoring blood flow within the first several hours. Rehabilitation strategies are widely used with limited success. The purpose of this study was to examine the effect of electrical stimulation on the impaired upper extremity to improve functional recovery after stroke. We developed a rodent model using an electrode cuff implant onto a single peripheral nerve (median nerve) of the paretic forelimb and applied daily electrical stimulation. The skilled forelimb reaching test was used to evaluate functional outcome after stroke and electrical stimulation. Anterograde axonal tracing from layer V pyramidal neurons with biotinylated dextran amine was done to evaluate the formation of new neuronal connections from the contralesional cortex to the deafferented spinal cord. Rats receiving electrical stimulation on the median nerve showed significant improvement in the skilled forelimb reaching test in comparison with stroke only and stroke with sham stimulation. Rats that received electrical stimulation also exhibited significant improvement in the latency to initiate adhesive removal from the impaired forelimb, indicating better sensory recovery. Furthermore, axonal tracing analysis showed a significant higher midline fiber crossing index in the cervical spinal cord of rats receiving electrical stimulation. Our results indicate that direct peripheral nerve stimulation leads to improved sensorimotor recovery in the stroke-impaired forelimb, and may be a useful approach to improve post-stroke deficits in human patients.

## Introduction

Stroke is the fifth leading cause of death in the U.S. and is the most common cause of adult disability. Nearly 4 million stroke survivors live with permanent neurological damage effecting their function and quality of life. Among them, 87% of strokes are ischemic in nature (1). Currently, the only approved treatments for acute ischemic stroke (tissue plasminogen activator and embolectomy) must be administered or performed in the early post-stroke period, which, unfortunately accounts for only 10% of all stroke patients as the rest do not reach a hospital early enough to receive such interventions (2–4). This leaves roughly 90% of ischemic stroke survivors to rely on rehabilitative therapies which show real but limited success in functional restoration. Among the many complications and sequelae of stroke, more than 75% of patients have upper extremity paresis (5) which includes the inability to properly use the thumb and fingers, as in a grasping motion (6, 7). Thus, improving upper extremity and hand function is of great importance in post-stroke rehabilitation and it is necessary to develop therapeutic approaches for this debilitating aspect of stroke recovery (8).

Neuromuscular electrical stimulation (NMES) has been used to facilitate rehabilitation post-stroke by improving and/or replacing volitional movement to enhance neural motor control, and hence reduce disability (9–11). NMES stimulates nerves to activate paretic muscles, and can be applied as either a supplementary tool combined with other rehabilitation strategies or used alone to improve functional recovery beyond the time of stimulation (12). The actual benefits of NMES are unclear as contradictory results from various clinical studies have emerged (11), and therefore more experimental studies are needed.

NMES can be used via invasive (implanted, percutaneous) or non-invasive (transcutaneous) devices (13, 14). For upper extremity rehabilitation, non-invasive approaches have been applied with conventional therapy in a cyclic pattern, EMG-triggered, or switch-triggered manner to facilitate activation of specific muscles to enhance task performance (15). However, the major issue of transcutaneous devices is that while a specific nerve of interest can be targeted, these devices also activate non-targeted nerves. Moreover, deeper nerves and muscles are more difficult to target using non-invasive techniques. Implanted devices (such as cuff electrodes) target specific nerves and reduce the current required to discharge the nerve while reducing non-targeted nerve activation (10, 16). While some implantable devices have been approved in Europe for use in aiding foot drop in stroke patients, none are currently FDA approved.

Previous studies have suggested that NMES may improve function by inducing neuroplastic changes in the spared motor cortex post-stroke (11, 14, 17). However, this possible cellular mechanism has not been fully investigated. Since changes in cortical efferent circuitry have been reported by our lab and others to play an important role in sensorimotor functional improvement after stroke (18–21), we sought to determine whether post-stroke NMES modulated neuroplasticity and improved function. In this report, we used a rat model of ischemic stroke to produce upper extremity paresis to assess whether peripheral nerve stimulation (*via* nerve cuff electrode) increased functional and volitional recovery in the effected forelimb.

## Methods

### Animal Subjects

All animal studies were approved by the Institutional Animal Care and Use Committee of the Edward Hines Jr. Veterans Affairs Hospital. A total of 24 male Long Evans black-hooded rats (2 months of age, HsdBLU:LE, Harlan Laboratories IN, Barrier 217) were used at the beginning of the study. Rats were housed in standard cages (2 rats per cage) with a 12-h light/dark cycle in a fully accredited animal care facility. Rats had access to fresh water and were fed standard laboratory chow (Harlan 2018 Rodent Diet). All rats were number coded by a laboratory member not participating in the study, and investigators conducting behavioral testing and neuroanatomical analyses were blinded to the treatment conditions throughout the experiments.

### Experimental Timeline and Overview

**Figure 1:**
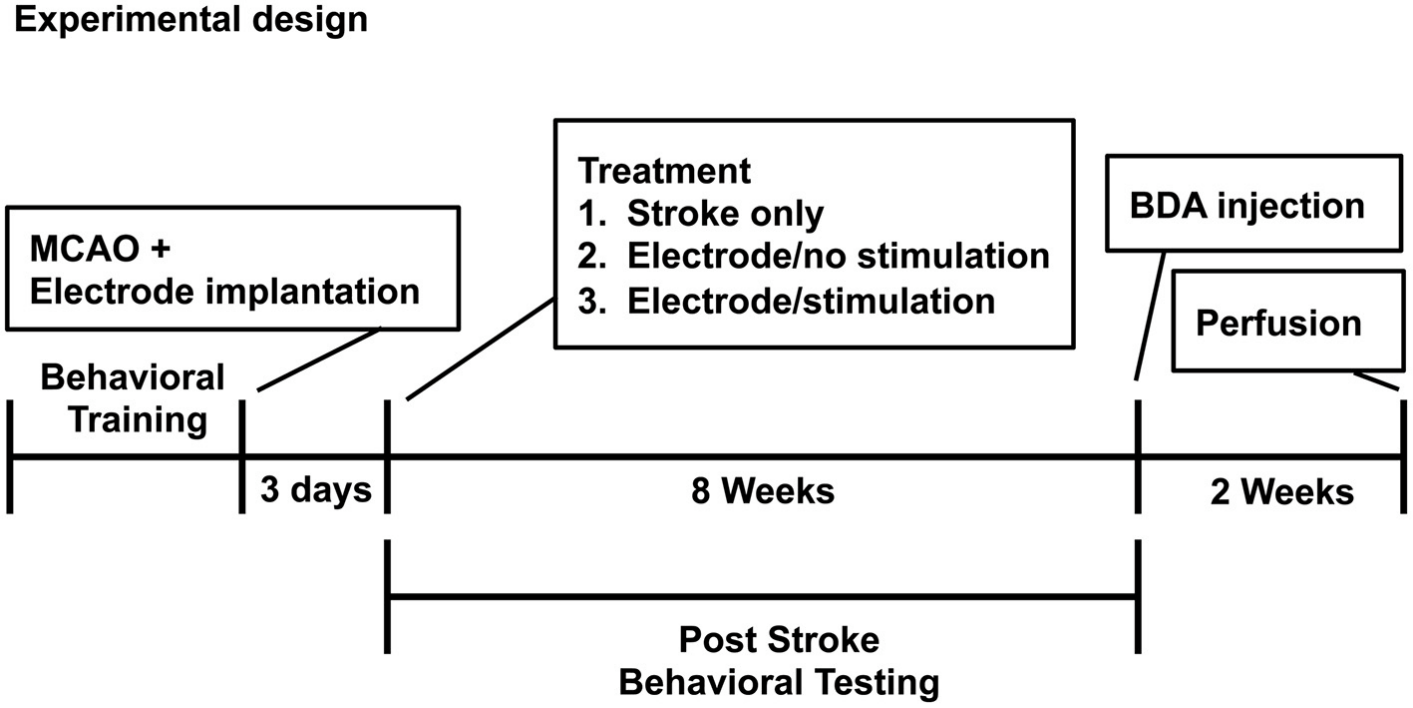
Experimental design and timeline.

The experimental timeline is shown in Figure 1.

### Behavioral Procedures

When the initial training period began, rats were reduced to 95% of their total body weight by food restriction; water was freely available. Behavioral tests were conducted at approximately the same time of day. Each animal was video recorded separately after the daily tests at baseline, 3 days following stroke, and at the end (8 weeks post-stroke) of the behavioral study. The recorded data was used to calibrate inter-rater variability and reaching error analysis.

#### Skilled Forelimb Reaching Test

This test was first performed as designed by Whishaw and our laboratory (22, 23) and used as described in our previous work (18, 20, 21, 24). It is well-established that the sensorimotor circuitry controlling motor control and related reaching and grasping of skilled reaching behavior is highly conserved in mammals (25). In brief, animals were placed in a transparent Plexiglas chamber (30 × 36 × 30 cm) with a rectangular opening (1.5 × 3 cm) in one wall. Small round food pellets (45 mg; Bilaney Consultants, Frenchtown, NJ) were placed one after the other onto the shelf at a distance of 1.5 cm from the opening. Rats were placed in the testing chamber daily until they learned to reach through the opening for the pellet, grasp the pellet and bring it to their mouth (a success). Rats were free to use either limb to reach through the opening and limb preference was determined and recorded for each animal. Rats reaching a baseline score with at least 16 successes out of 20 pellets per day for three consecutive days were enrolled in the study for stroke surgery. A maximum time limit of 5 min per session was given. Each rat resumed testing 3 days after stroke, and then daily (Mon-Fri) until the end of the study. The parameters recorded for the skilled forelimb reaching test included (1) the amount of time needed to obtain all 20 pellets, and (2) the success score, i.e., the number of pellets grasped with the preferred limb and placed into the mouth.

#### Reaching Error Analysis

Each animal was video recorded after the daily tests at baseline, 3 days following stroke, and at the end (8 weeks post-op). Reaching video files were analyzed frame by frame using QuickTime Player. Each reach error was recorded during analysis as described (Table 1). Descriptions included (a) the outcome (success or failure) and (b) description of event (short reach, inaccurate aim, bad grasp, etc.). From the described attempts four overarching categories emerged (miss, touch, drop, and success) with two subtypes per category. Categories were assigned a numerical value based on a progression of a reach (1–8). Therefore, rats that were able to grasp received a higher numeric value than rats that could not extend their limb.

**Table 1:** Reaching Error Analysis.

**Miss**	**Touch**	**Drop**	**Success**
*1 – No Extension* • Limb contracted • Limb did not extend past window or just past • Curled wrist and digits	*3 – No grasp* • Limb extended past window • Paw and digits are flat or curved toward body • Paw planted flat on pellet – digits do not curl around pellet • Pellet pulled through window or paw lifted up and back	*5 – Early Drop* • Limb extended past window • Paw and digits slightly angled toward pellet • Digits are planted and curled around pellet • Pellet is in paw as limb is retracted • Paw is not brought to mouth but placed on ground near starting position • Pellet falls out of paw while the limb is on the ground or while another reach is attempted	*7 – Partial Success* • Limb extended past window • Paw and digits slightly angled toward pellet • Digits are planted and curled around pellet • Pellet is in paw as limb is retracted • Paw is brought to mouth • Digits are straightened as paw is brought to mouth. Removing pellet from grasp requires multiple licks.
*2 – Poor aim* • Limb extended past window • Digits curled in grasp motion in air above pellet • Limb/paw ended on either side of pellet and grasps	*4 – Inaccurate grasp* • Limb extended past window • Paw and digits are flat or curved • Digits planted on and curled around pellet • During limb retraction, pellet is either dragged or immediately propelled away from between digits	*6 – Late Drop* • Limb extended past window • Paw and digits slightly angled toward pellet • Digits are planted and curled around pellet • Pellet is in paw as limb is retracted • Paw is brought to mouth • Pellet falls while attempting to remove from grasp	*8 – Complete success* • Limb extended past window • Paw and digits slightly angled toward pellet • Digits are planted and curled around pellet • Pellet is in paw as limb is retracted • Paw is brought to mouth • Digits are straightened as pellet is placed in mouth

#### Bilateral Tactile Adhesive Removal Task

This task has been shown to be sensitive for the detection of somatosensory deficits following sensorimotor cortex injury (26, 27). Small round adhesive stickers (Creatology adhesive foam sheet, 0.7 mm diameter, Michaels, Irving, TX) were placed onto the plantar surface of both forepaws. The order of contact, contact latency, and removal latency for each paw was recorded independently for each limb. Each rat was given maximal 120 s to complete initiation and removal of the adhesive. One hundred twenty seconds was recorded if the rat did not complete the task in time.

### Stroke Surgery

A focal ischemic stroke was induced via middle cerebral artery occlusion (MCAO) modified from Chen et al. (28) and as in our previous work (18, 20, 21, 24). Rats were anesthetized with isoflurane (2–3% in oxygen) and core body temperature was maintained between 36.5 and 37.5°C during the entire procedure. The bilateral common carotid arteries (CCA) were first isolated from a ventral longitudinal cervical incision and the common carotid artery ipsilateral to the hemisphere corresponding to the preferred forelimb was permanently ligated. Rats were then placed in a stereotaxic frame and a vertical incision was made between the eye and ear. The temporalis muscle was retracted, and a burr hole was made to expose the middle cerebral artery (MCA) on the opposite side of the preferred limb, which was subsequently ligated with a 10-0 suture and then transected with microscissors. The contralateral CCA was temporarily occluded for 45 min using an aneurysm clip. After removal of the aneurysm clip, all wounds were closed and rats were either kept warm until awake or were kept anesthetized for nerve electrode cuff implantation. Rats were monitored during the post-op period until fully awake and able to eat and drink.

### Nerve Cuff Electrode Placement

**Figure 2:**
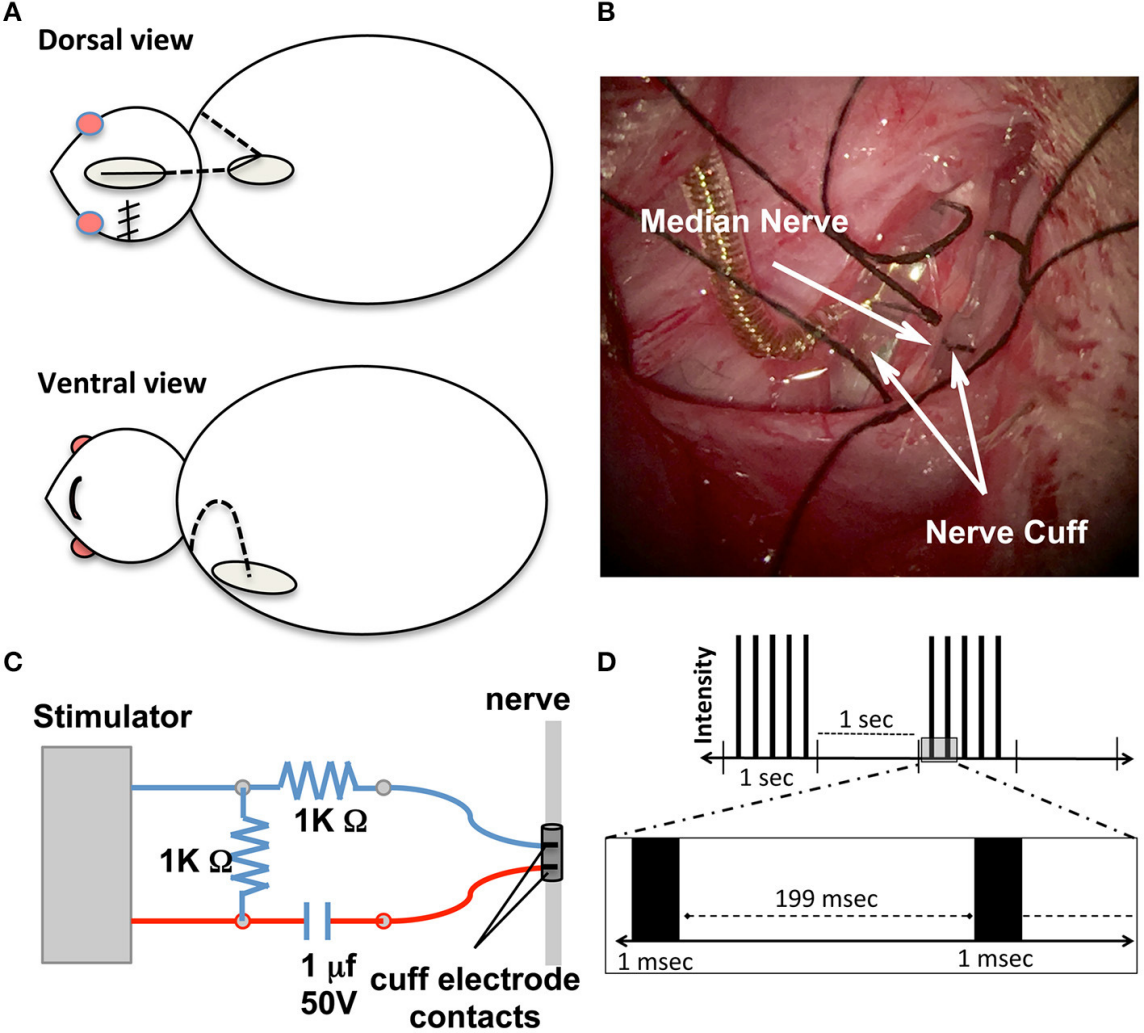
Nerve cuff placement. **(A)** Schematic of surgical procedures for nerve cuff placement. Dotted lines represent the lead wire and ovals represent surgical incision sites. **(B)** Intraoperative photographs of the cuff enclosing the nerve according to the experimental design. Gold colored coil is the lead wire leaving the cuff. Black sutures were tied to keep cuff secure around nerve. **(C)** Schematic diagram of electrical stimulation circuit setups. The stimulator is the power source and **(D)** Schematic demonstration of stimulation parameters. Each black bar represents an individual pulse segment.

Placement of the electrode cuff (MicroProbes for Life Science, Gaithersburg, MD) was performed immediately after completion of MCAO under the same anesthesia (Figure 2). The procedure was adapted from Cho et al. (29) as shown in Figures 2A,B. Skin incisions were made on the top of the head and at the interscapular region. Additionally, an incision was made along the upper medial aspect of the impaired extremity and extended into the axilla to expose the pectoralis major muscle. The muscle was then retracted to expose the brachial plexus and the median nerve was isolated. A tunnel under the skin was created from the top of the head to the interscapular incision and to the axilla, and the electrode wire was guided into the axilla opening. The median nerve was placed in the electrode cuff and stabilized by sutures (Figure 2B). The cuff was custom made with the following specifications: 0.75 mm inner diameter, two 100 μm platinum contacts were situated 1 mm longitudinally apart and 2 mm to the edge of the cuff, an ultra fine lead leaving the cuff at a 45 degree angle with a total silicone encapsulation running for 100 mm, and connected to an *in vivo* 1 MS303-120 right-angled two channel connector (PI Technology, Roanoke, VA). Partial loops of the lead were left in the neck wound to allow for movement and to prevent strain on the electrode/nerve. A small guide hole was placed in the skull frontal bone followed by a stainless steel screw placement. The connector and screw were then covered with a fast-setting dental cement to form a connector cap. The electrical continuity of the electrode leads was tested using an ohmmeter. The head incision was sutured while leaving the top part of the connector cap above the skin and exposed to allow for connection to the stimulator.

### Peripheral Neuromuscular Electrical Stimulation

A schematic of the electrical stimulation circuitry is shown in Figure 2C. Charge balanced stimulation was used to prevent corrosion of the electrodes and deleterious effects on the nerve. The capacitor coupled stimulation balanced the stimulation current during the pulse with an equal and opposite discharge during the inter-pulse period. Rats undergoing stimulation were individually placed into a repurposed forelimb reaching box after their electrode was connected to the stimulator. The leads from the electrode to the stimulator were attached to the roof of the box so the rat could move freely. Using a Grass S11 constant voltage stimulator, stimulation voltage was gradually increased until muscle contraction was felt (deemed threshold level). A 1.5-fold increase over the threshold voltage was applied to induce muscle contraction throughout the 30 min stimulation sessions. Threshold current was determined by measuring the voltage across a 1 K Ohm resistor in series with the stimulating electrode and using Ohm's law to solve for current. Threshold currents were recorded after completion of the electrode cuff implantation. The stimulation parameters were adapted from our previous work on a spinal cord injury model with modifications (30). Other parameters included 1.0 ms in duration of stimulation, delivered every 2 s in 1-s trains of 5 Hz (Figure 2D). Daily 30 min stimulation sessions began 3 days after MCAO surgery and continued (Monday–Friday) for a total of 8 weeks. Rats in the Stroke/No E-stim group had the exact same placement of the electrode post-stroke, were placed in the stimulation boxes for the same time period, but did not receive any electrical stimulation. Each stimulation session occurred at least 2 h after the behavioral tests. The staff applying stimulation was different from the staff testing animals.

### Biotin Dextran Amine Tracing

When behavioral testing was complete, rats were re-anesthetized with isoflurane (2–3% in oxygen) and secured in a stereotaxic frame. A 2 mm burr hole was made to expose the sensorimotor cortex of the hemisphere opposite to the stroke lesion site. Two 1 μl aliquots of 10% BDA solution in 0.01 M phosphate buffer (pH 7.2) were injected into the caudal forelimb motor cortex (coordinates: 0.5 mm caudal/2.5 mm lateral, 1.0 mm caudal/3.0 mm lateral to bregma, and both at 1.5 mm ventral from the brain surface), as defined electrophysiologically by Neafsey et al. (31).

### BDA Histochemistry

BDA histochemistry was performed as previously described by our laboratory (18, 20, 32). Two weeks after BDA injections, rats were euthanized with sodium pentobarbital (100 mg/kg; i.p.) and perfused transcardially with heparinized PBS solution followed by fixative [4% paraformaldehyde (PFA) in 0.1 M phosphate buffer]. The brains and spinal cords were removed, post-fixed, cryoprotected, and frozen sectioned coronally at 40 μm. Sections were collected every 200 μm in one series. Endogenous peroxidases were blocked via incubation with 0.1% hydrogen peroxide solution. Sections were then washed and incubated overnight with an avidin-biotin-peroxidase complex (ABC elite, Vector Labs). The sections were washed again and pre-incubated with 0.4% ammonium nickel sulfate followed by 0.015% 3.3-diaminobenzidine (DAB), and finally reacted with 0.004% hydrogen peroxide. The reaction was stopped by washing with tris buffer and the sections were left to air dry before being lightly counterstained with Nissl stain and coverslipped.

### Stroke Size Analysis

Nissl-stained coronal brain sections were quantitatively analyzed every 400 μm between +4.7 to −5.2 mm from bregma according to Paxinos and Watson (33) using a computer-interfaced imaging system (Image J, NIH, Bethesda, MD) as described previously (18, 20, 21, 34). Stroke size was expressed as a percent of the intact contralateral hemispheric area (total area of the intact contralateral hemisphere minus total area of the ipsilesional hemisphere divided by the total area of the intact contralateral hemisphere).

### Neuroanatomical Analysis

The analyses for corticospinal plasticity described in this section were performed as previously reported (18, 35). All analyses were done on coded slides so that the investigator was blinded as to the experimental group. Anatomical structures were identified with the atlas of Paxinos and Watson (33).

#### Quantification of Corticospinal Tract Labeling Crossing the Midline and in the Dorsal Column

BDA-labeled fibers in the dorsal column were counted using stereological methods (StereoInvestigator, MBF Bioscience, Williston, VT) on the first two C5 sections analyzed for midline fiber crossing. In brief, dorsal column outline tracings were performed under a 2.5X objective. Both the sampling grid size (75 × 75 μm) and the sampling ratio (20%) were optimized to provide adequate sampling of the entire dorsal column. The estimated total BDA-labeled CST fibers of both sections were averaged as an internal reference for BDA injections and staining. This was done in order to standardize the total number of labeled midline crossing fibers in the spinal cord (18).

On sections analyzed, all BDA-positive fibers crossing the midline were counted under a 40X objective. Twenty alternate sections (400 μm apart) at the cervical spinal cord levels C5–C8 were totaled. To correct for inter-animal tracing variability, the total number of crossing fibers was divided by the number of labeled corticospinal fibers in the dorsal column (as determined above) multiplied × 10^4^ and expressed as fibers crossing the midline per ten thousand labeled corticospinal axons.

### Inclusion/Exclusion Criteria

#### Skilled Forelimb Reaching Test

Prior to surgery, animals were excluded from the study if they were unable to successfully reach for 16 out of 20 pellets within 5 min after 3 weeks of training on the task. Three days post-stroke, all rats were tested. Animals showing severe deficits were excluded from the study. Severe deficits were defined as the inability of a rat to reach using his impaired forelimb through the open window for at least one out of 20 attempts. Conversely, if a rat showed only a mild deficit 3 days post-stroke, which we defined as reaching successfully for 10 or more pellets out of the 20 attempts, they were excluded from the entire study (21).

#### Bilateral Tactile Adhesive Removal Task

In order for the handling of the animal to not interfere with the subcutaneous leads and the electrode cuff, animals which struggled excessively during the adhesive removal task at post-op day three were excluded only from this analysis.

#### Stroke Lesion

Following our previous reports (18, 21), rats were excluded from the study if lesion analysis did not show sensorimotor cortex involvement and/or showed extensive dorsal lateral (DL) striatum involvement.

#### BDA Labeling

For this analysis, rats demonstrating dorsal column BDA-labeled corticospinal fiber counts of <500 were excluded from crossing fiber analysis due to poor BDA transport or insufficient injection site uptake.

### Statistical Analysis

Analyses of all data were performed with SPSS version 13.0 (SPSS Inc., Chicago, IL). A 2-WAY repeated measure ANOVA (time × treatment) was used to analyze differences in the forelimb reaching test between three experimental groups, followed by Tukey *post-hoc* to compare weekly mean values between the three experimental groups. The adhesive removal test was analyzed using Non-parametric Mann-Whitney test. Spinal cord midline crossing index were analyzed using a Student's *T*-test. Forelimb reaching results were correlated with spinal cord midline crossing index. A *p* value ≤ 0.05 was considered significant. All error bars shown are ±SEM. All bar graphs show individual data points (*n* value).

## Results

At the beginning of the study, 26 animals received skilled forelimb reaching task training. Three animals were removed (*n* = three) from the study prior to surgery due to failing to reach baseline, i.e., they were not successful at grasping at least 16 out of 20 pellets within 5 min. A total of five animals (*n* = 5) were further excluded from the study 3 days after stroke. Of those, three were removed due to the inability to reach for the pellets (severe deficit), and two were removed because they showed only a mild reaching deficit.

At the completion of electrode cuff implantation, the average threshold currents (mean ± SEM) were 0.11 ± 0.01 mA.

### Stroke Lesion in the Sensorimotor Cortex

**Figure 3:**
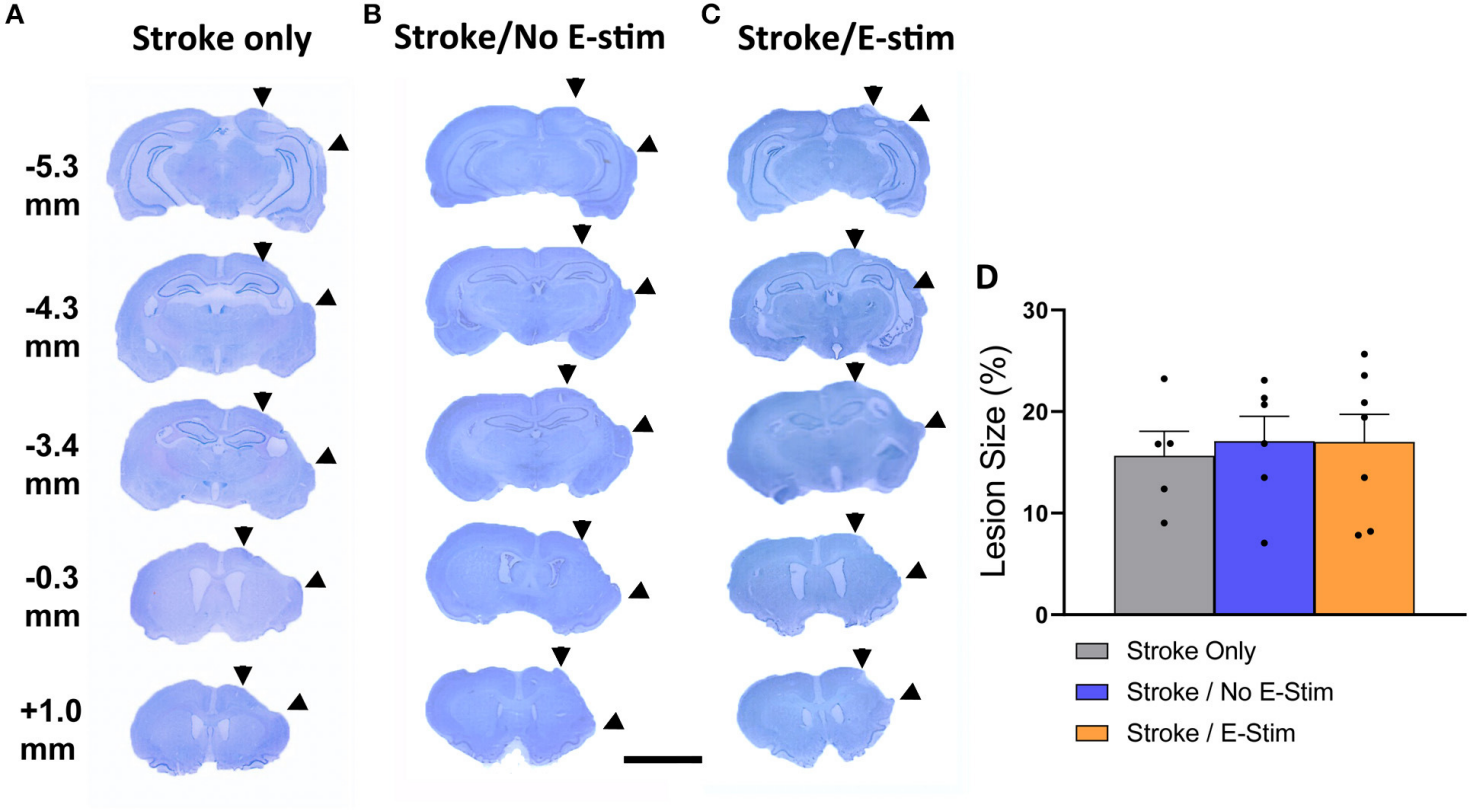
Lesion analysis. Nissl stain sections and their relative distance from bregma showing typical ischemic lesion of all three groups of rats **(A–C)**. Area between two arrowheads on each brain section indicates stroke-induced necrotic regions. The numbers indicate relative distance to bregma. The scale bar indicates 0.5 cm. **(D)** Lesion analysis showed no difference in the lesion size between groups (Stroke only vs. Stroke/No e-stim vs. Stroke/E-stim ANOVA, *p* > 0.05).

Stroke lesions in all rats showed ipsilateral sensorimotor cortex necrosis with no or minimal anterior striatum involvement and without other anatomical structure damage (Figures 3A–C). There was no statistical difference in stroke size between groups (Stroke only, 15.67% ± 2.4 SEM; Stroke/No E-stim, 17.09% ± 2.45 SEM; Stroke/E-stim, 17.02% ± 2.73 SEM) (one-way ANOVA, *p* > 0.05) (Figure 3D).

### Functional Improvement After Direct Peripheral Nerve Electrical Stimulation

#### Skilled Forelimb Reaching

**Figure 4:**
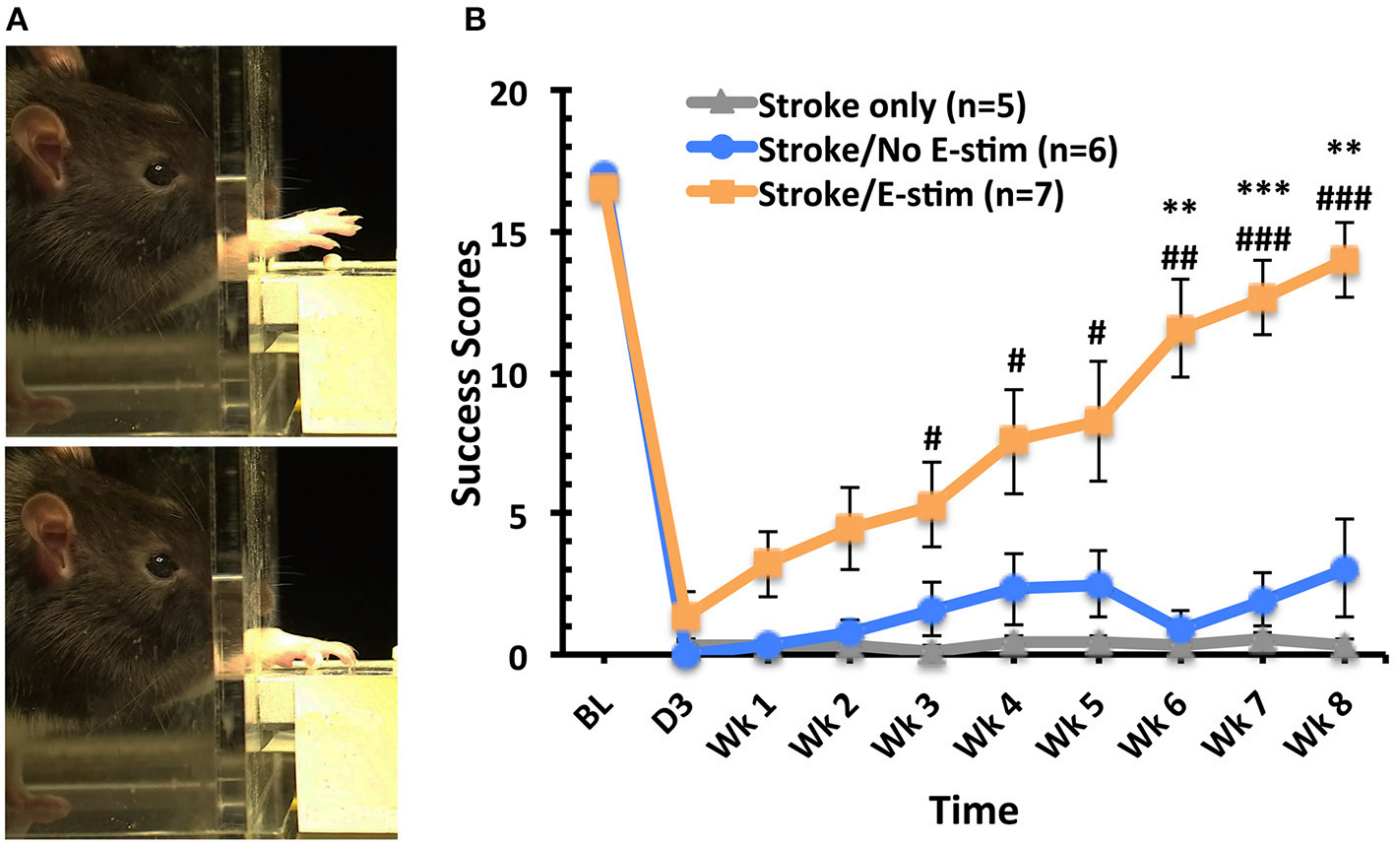
Skilled forelimb reaching task analysis. **(A)** Sequential frames of the skilled forelimb reaching task performed by an adult rat grasping a sugar pellet. **(B)** Success scores for reaching task (out of 20 reaches) across time. No difference between groups at baseline. Marked deficits in successfully obtaining pellets with the stroke-impaired limb were apparent 3 days post-op (before treatment) with no significant difference between groups. Rats receiving electrical stimulation showed significant overall improvement of the skilled forelimb reaching test when compared to stroke only and stroke with sham stimulation (two way repeated-measure ANOVA, main effect time *p* < 0.0001, main effect treatment *p* < 0.0001, interaction *p* < 0.001). Weekly comparison showed that animals receiving electrical stimulation exhibited significant improvement in the success scores than the Stroke only group (#*p* < 0.05 at week 3, 4, and 5, ##*p* < 0.01 at week 6, ###*p* < 0.001 at week 7 and week 8) and Stroke/No e-stim group (**p < 0.01 at week 6 and 8, ***p < 0.001 at week 7) and the improvement persisted until the end of the study. There was no difference between two control groups at any time point (Tukey's *post hoc* analysis, error bars indicates ±SEM).

After training on the skilled reaching task, baseline success scores showed no significant difference between the three groups (Figure 4). Three days post-MCAO, all rats exhibited a marked deficit in reaching success using their preferred limb with no difference between groups. However, rats receiving electrical stimulation showed significant overall improvement of the skilled forelimb reaching test when compared to stroke only and stroke with sham stimulation (two way repeated-measure ANOVA, main effect time *p* < 0.0001, main effect treatment *p* < 0.0001, interaction *p* < 0.001). Differences in reaching success scores were first apparent 3 weeks post-MCAO between stroke only animals and those receiving electrical stimulation (Tukey, *p* < 0.05). This significant difference between electrical stimulation and stroke only groups continued throughout the remaining weeks (*p* < 0.05 at week 3, 4, and 5, *p* < 0.01 at week 6, *p* < 0.001 at week 7 and week 8). Six weeks after MCAO, those rats receiving stimulation showed significant increases in reaching success compared to those with sham stimulation (*p* < 0.01). This significant increase persisted throughout the remainder of the behavioral testing (*p* < 0.001 at week 7, *p* < 0.01 at week 8). Additionally, the reaching success score of animals receiving electrical stimulation was not significantly different from baseline scores at 8 weeks, with stimulated animals receiving, on average, 83% of the baseline score.

#### Reaching Error Analysis

**Figure 5:**
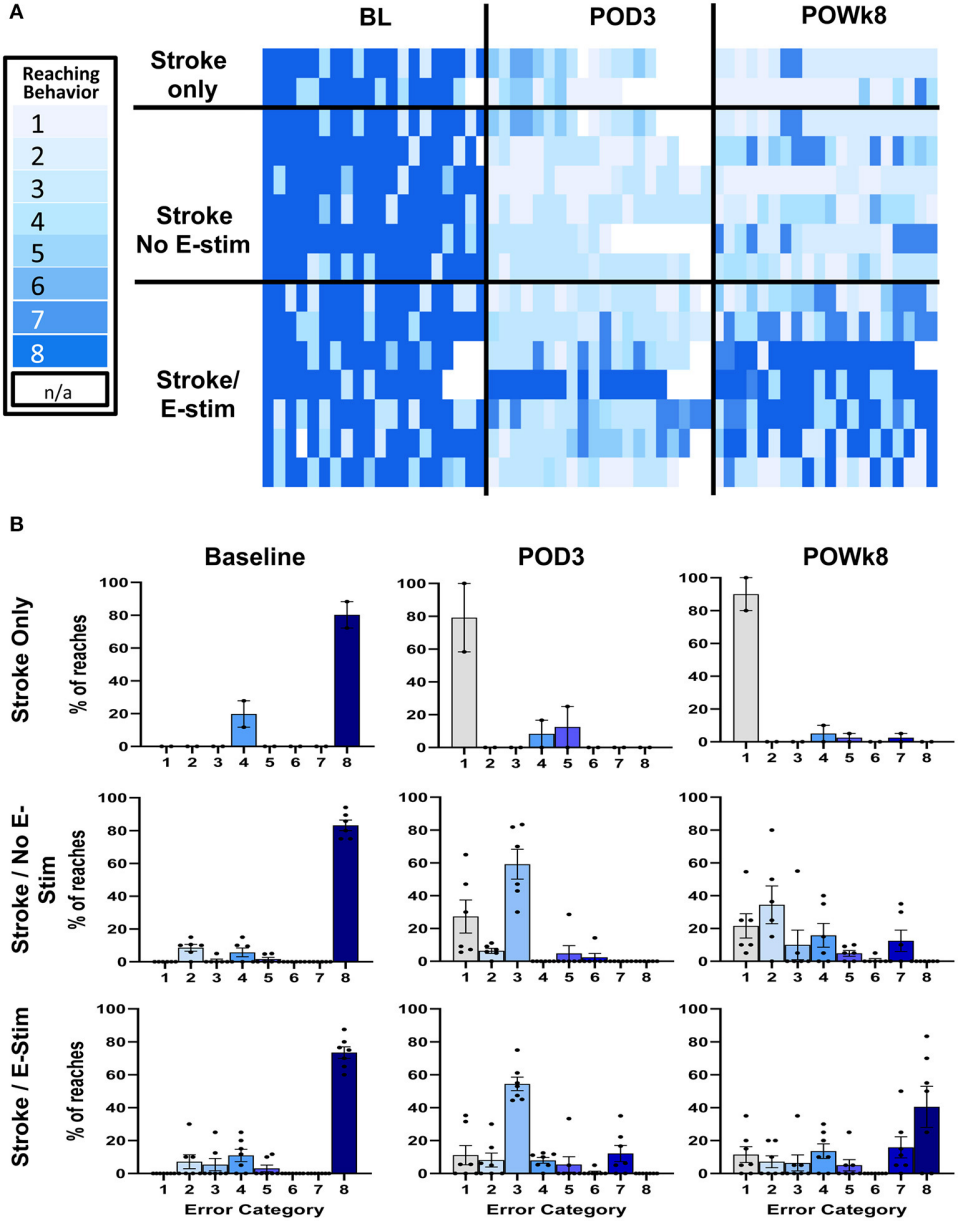
Reaching error analysis. **(A)** Reaching error analysis of individual animals in each experimental group. All animals' reaching behaviors were recorded and analyzed at pre-stroke baseline, 3 days post-op before stimulation, and at the end of 8 weeks before BDA injection. The errors were categorized based on reaching error type. Each trial was composed of 20 different reaches where each reach was categorized independently. Reaching behaviors are categorized as follows and range from the lightest blue to the darkest blue: (1) Miss – no extension, (2) Miss – poor aim, (3) Touch –no grasp, (4) Touch – inaccurate grasp, (5) Grasp – dropped early, (6) Grasp – dropped during eating, (7) Success – arm motions not smooth, and (8) Total Success. White boxes represent missing data points (N/A). **(B)** Bar graph plots of reaching errors. At baseline, most reaches were successes across groups. However, of the unsuccessful reaches, most were in the moderate severity categories. At 3 days post-op (POD3), animals across experimental groups committed errors in the more severe error categories (i.e., 1–3) with less complete success. At the end of behavioral studies (POWk8), animals receiving E-stim not only improved in the number of successful reaches but also committed less errors in the more severe categories. In the non-stimulated groups, animals either showed no improvement (Stroke only) or there was a slightly right shift toward the less severe error categories without increasing success (Stroke/No E-stim).

Qualitative analysis of the forelimb reaching errors showed a decrease in deficit severity across time that was particularly obvious in rats receiving E-Stim (Figure 5). Reaching behaviors were analyzed at pre-stroke baseline, 3 days post-stroke before stimulation, and at the end of 8 weeks just prior to BDA injection. Recordings of three animals in the Stroke only group were dropped from the analysis due to poor recording quality. At 3 days post-stroke, errors were comprised mainly of missed pellets or failure to grasp the pellets. Rats receiving either no stimulation or sham stimulation remained similar in the error types at the end of the study. On the contrary, rats receiving E-stim showed similar errors as the other two control groups at 3 days post-stroke, but improved to less severe categories by the end of the study (Figures 5A,B), indicating that not only did electrical stimulation improve overall success in obtaining pellets, but also lessened the impact of the stroke by decreasing the severity of the errors that did persist.

#### Bilateral Tactile Adhesive Removal Task

**Figure 6:**
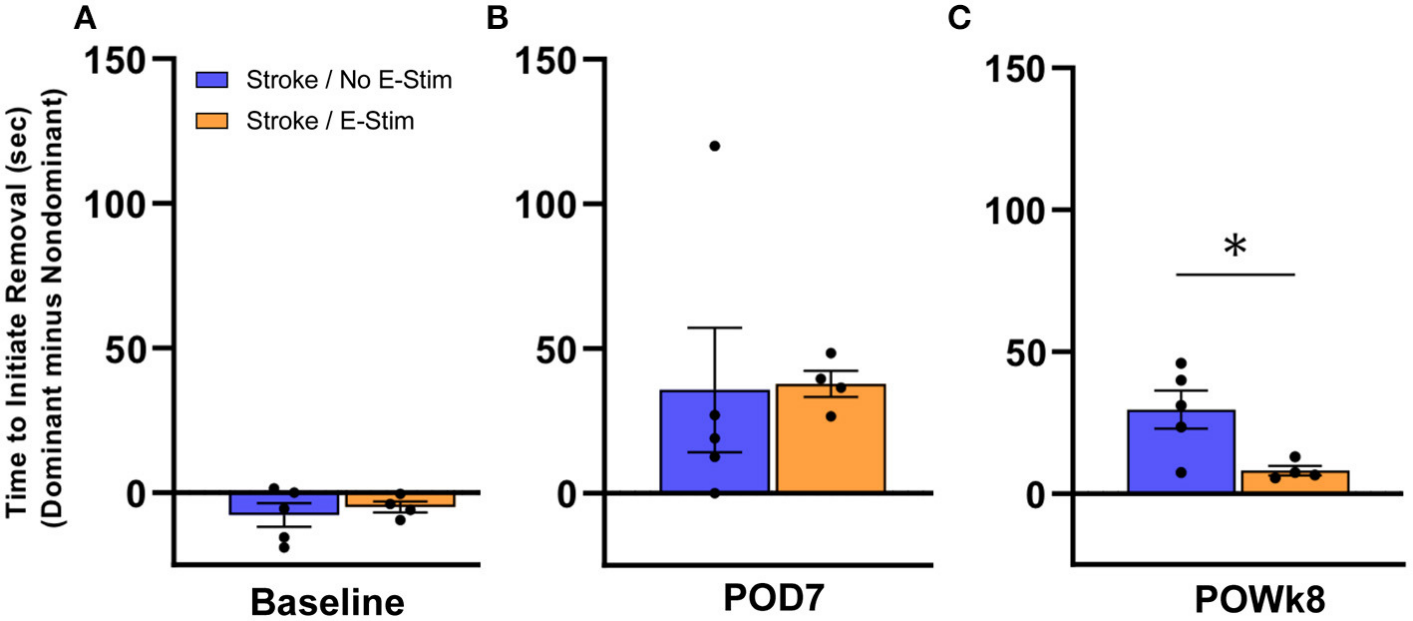
Adhesive removal analysis. Both Stroke/No E-stim and Stroke/E-stim groups showed similar time in the initiation difference between two forelimbs for adhesive removal at pre-stroke baseline **(A)** and such initiation difference increased greatly at 7 days post-stroke **(B)** (*p* > 0.05). **(C)** At the end of behavioral tests, i.e., 8 weeks after stroke, animals receiving direct electrical stimulation on median nerve showed significant less time difference between two limbs to initiate adhesive removal (mean ± SEM, Non-parametric Mann-Whitney test, *p < 0.05).

In order to examine whether electrical stimulation to the median nerve in the forelimb affected sensory perception, we performed the adhesive removal test on rats that received implantation of an electrode (Figure 6). As this test required a longer duration of handling, animals that showed excessive struggle/agitation were excluded from this analysis in order to preserve and not disturb electrode placement. Sensory perception was analyzed using the difference in time it took for the rat to initiate removal of the adhesive sticker from its stroke-affected forepaw compared to its other forepaw. At baseline testing prior to stroke (Figure 6A), no difference in latency to initiate removal was observed between the Stroke/no E-stim group (*n* = 5) and the Stroke/E-stim group (*n* = 4). One week post-stroke (Figure 6B), the difference in latency to initiate removal between dominant and non-dominant forepaws was increased in both Stroke/no E-stim (mean = 35.7 s) and Stroke/E-stim (mean = 37.8 s) group, indicating a sensory deficit caused by the stroke. However, at 8 weeks post-stroke (Figure 6C), rats that received the E-stim therapy showed a significant decrease in latency to initiate between impaired and non-impaired forepaws (mean = 8.1 s) compared to the rats that only received sham stimulation (mean = 30 s) (nonparametric Mann-Whitney test, *p* < 0.05), suggesting that electrical stimulation contributed to improved sensory perception in the stroke-affected limb.

### Quantification of Neuronal Plasticity

**Figure 7:**
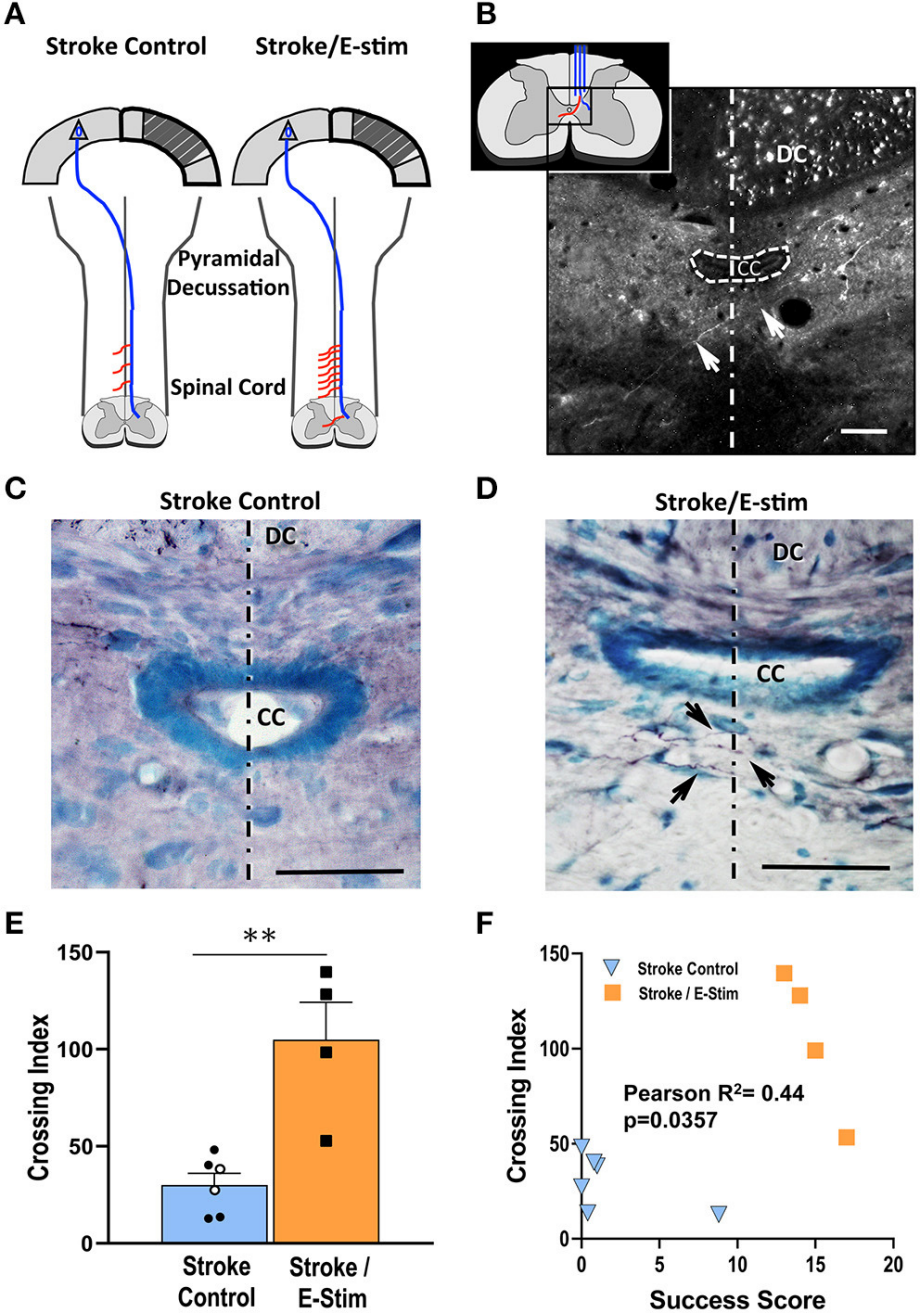
Neuroanatomical analysis. **(A)** Schematic diagram showing corticospinal plasticity presented as more midline crossing fibers (red lines) along the cervical enlargement after stroke and E-stim treatment. Diagram adapted from Wahl et al. (36). **(B)** An enlarged schematic midline fiber crossing diagram (inset) and a representative fluorescent photomicrograph shows an alexa fluor 488 streptavidin-labeled BDA-positive midline-crossing corticospinal fiber (arrows) at C6 level. The fluorescent dots in the right dorsal column are BDA-positive corticospinal fibers. **(C,D)** Photomicrographs of BDA-reacted C6 spinal level sections show differences of corticospinal midline crossing fibers (arrows) between a Stroke Control **(C)** and Stroke/E-stim animals **(D)**. **(E)** Stroke/E-stim treated animals exhibited a significant higher midline crossing corticospinal fiber index in comparison with Stroke control (pooled Stroke Only and Stroke/No E-stim animals) (mean ± SEM, **p < 0.01, Student's *t*-test). Black circles represent Stroke/No E-stim and white circles represent Stroke Only animals. **(F)**. Midline crossing corticospinal fibers showed positive correlation with the final forelimb reaching success score across 3 groups of animals (*P* < 0.05). The dotted lines indicate the midline. The scale bar indicates 50 μm. DC, dorsal column; CC, central canal.

Compensatory sprouting and neuroanatomical plasticity were analyzed for corticospinal midline crossing fibers originating from the contralesional forelimb cortex using the anterograde neuroanatomical tracer BDA (Figures 7A,B). Due to exclusion criteria requiring at least a count of 500 dorsal column BDA-labeled corticospinal fibers, a total of 8 rats (Stroke Only = 3, Stroke/No E-stim = 2, and Stroke/E-stim = 3) were removed from this analysis due to poor BDA transport or insufficient injection site uptake. Since the two control groups showed no statistical difference in crossing fibers, they were pooled as Stroke Control for analysis (Figure 7C).

Rats receiving E-stim therapy (Figure 7D) exhibited nearly three times the midline crossing index (104.97 ± 19.22 SEM) in the cervical spinal cord as compared to the combined Stroke control group (32.52 ± 5.73 SEM) (Figure 7E, *p* < 0.05, Student's *t*-test). Further analysis (Figure 7F) showed that there was a direct correlation between the corticospinal fiber midline crossing index and the final forelimb reaching score for each rat (*r* = 0.44, *p* < 0.05), suggesting a positive relationship between the number of fibers that crossed the midline and functional recovery.

## Discussion

Our results show that daily, direct median nerve stimulation to the stroke-impaired forelimb in the adult rat led to significant improvement in the skilled forelimb reaching test, and also reduced the time to initiate adhesive removal on the stroke-impaired limb, indicating significant sensorimotor recovery. Furthermore, this therapy enhanced cortico-efferent plasticity from the contralesional cortex to the deafferented spinal cord, which may be the underlying substrate for the observed improvement in function.

One of the main goals for stroke survivors is improving hand and finger use of the paretic upper extremity, such as flexion/extension and adduction/abduction motion. The median nerve was directly targeted in this study as it innervates muscles of the forearm and digits one through four. Furthermore, there are great similarities between humans and rats in performing reaching movements that largely involve the flexion movements that are controlled by the median nerve (37–39). As a mixed nerve, the median nerve also transmits sensory information from the palm and some of the digits (39). Our results showed that direct median nerve stimulation improved performance in a sensorimotor based (skilled reaching task) and a sensory (adhesive removal) task. In the skilled forelimb reaching task, we observed that rats receiving E-stim improved up to 80% or better performance of their baseline values, compared to those who did not receive E-stim. Further analysis of errors leading to non-successful reaches showed that in all three groups rats demonstrated similar types of errors at baseline, which is to be expected since they were essentially “normal” rats. At 3 days post-stroke, and before E-stim was started, the same types of errors were demonstrated across all groups. These errors largely were categorized in the more severe error categories, i.e., inability to aim properly or grasp with their digits. However, there was an exception to this pattern. One rat in the E-stim group did not make as severe of errors. This rat was not excluded from the study as its lesion size and performance on the daily reaching task post-stroke did not differ significantly from the rest of the group.

At the end of the 8 weeks, rats receiving E-stim showed a marked shift in their errors toward categories considered less severe, compared to the stroke/No E-stim group whose errors were predominantly still classified in the more severe categories. This indicated not only an improvement in the total success scores from the reaching task, but a decrease in the overall severity of the stroke impact on forelimb function. Several reports have shown that peripheral electrical stimulation induced neuroplastic changes in the motor cortex (see Chipchase et al. for review) (40), in particular when the stimulation intensity was above the motor threshold (41, 42). Peripheral stimulation of 1.5–2 times the motor threshold (as was used in this study) not only induced a response in the corresponding cortex, but also the contralesional cortex (43–45). This bilateral activation of the cortex may play important roles for cortical reorganization, as the homotopic contralesional cortex is a major site for cortical plasticity leading to functional recovery (20, 46–50).

Our laboratory and others have reported that improved functional outcomes after CNS injury can be induced through a variety of approaches (i.e., neurite inhibitors, pharmacologic interventions, environmental enrichment, stem cells) (18, 51–55). Furthermore, it has been shown that peripheral nerve stimulation induced neuroplastic changes (40), therefore, we sought to determine if E-stim therapy led to new corticospinal connections from the contralesional cortex (47, 56). BDA analysis in the present study revealed that rats which received E-stim had increased spinal cord midline crossings of corticospinal tract axons originating in the contralesional motor cortex and terminating in deafferented cervical spinal areas. Moreover, by plotting both reaching score and fiber crossing index, two distinct treatment-based populations were evident (Figure 7F), which supports the significance of neuroplasticity in functional recovery. This finding is consistent with our previous studies demonstrating that remodeled motor pathways at the spinal cord level correlated with improved function after cortical lesions (18, 35). Although plastic changes in the contralesional cortex may serve as the origin of improved functional outcomes, the role of the perilesional cortex cannot be ignored (57–59). Studies have shown that enhanced synaptic efficacy and either an increase or reorganization of axodendritic synaptic density of the perilesional cortex is also closely associated with spontaneous functional improvement after stroke (60). However, larger stroke size in our model may lessen the availability of intact perilesional cortex in the forelimb area. Further studies will be needed to examine whether anatomical plasticity occurs in response to peripheral electrical stimulation in the perilesional region.

Only young adult animals were examined in the current study (2 months of age at the start of the study). Our previous studies in aged rats showed that improvement in sensorimotor performance (19) and spatial memory (61) can still be achieved post-stroke with the same interventions utilized in young adult rats (18, 24, 32). Therefore, we expect the same approach in this study will lead to similar outcomes in the aged rats. However, we may observe a different recovery pattern, such as delayed recovery (19).

Due to the lack of standard parameters, such as time to start stimulation after stroke and stimulation frequency/intensity, the beneficial effects of NMES on stroke patients cannot be verified easily. The stimulation parameters chosen in the present study were based on our previous report on spinal cord injury in the rat (30). Several clinical studies applying NMES alone suggested sensorimotor function improvement in stroke patients (62, 63) although with stimulation frequencies between 10 and 50 Hz. Further, a report showed that activation at 5 Hz on the cortex did improve stroke patients' motor function (64). Our present study using a lower frequency at 5 Hz is supportive of those clinical studies showing that NMES has great potential to be used alone as an option to promote functional improvement in stroke patients. More importantly, our model can be used to further systemically explore the therapeutic effects of various parameter combinations.

To our knowledge, there is no known research on cellular mechanisms involved in implanted NMES in stroke animals. However, we have considered several possible mechanisms that may contribute to the corticospinal plasticity presented here. (1) Peripheral nerve stimulation could recruit contralesional and surviving ipsilesional cortical neurons to undergo axonal remodeling and motor map reorganization (14, 65–67). (2) Peripheral nerve stimulation could affect all types of neural pathways including sensory, motor, proprioceptive, as well as polysynaptic transmission (44, 45, 63). In this way, future studies should investigate whether median nerve E-stim in this stroke model leads to plastic changes or modulation of structures along the ascending pathway, such as neural modulation in the periaqueductal gray (68). (3) In addition to the plasticity enhancing trophic factors brain-derived neurotrophic factor (BDNF), neurotrophin-3 (NT-3), and cytokines (tumor necrosis factor-alpha, interleukin-6) induced in the spinal cord after stroke (69), plasticity at the spinal cord level may be further enhanced through stimulation-induced changes of the micro-environment such as release of signaling molecules, BDNF (70) and/or insulin-like growth father (IGF) in muscle and spinal cord (71). These trophic factors are known to promote neural growth and enhance neuronal plasticity. Using this model, these trophic factors, and others, should be assayed to determine if changes in their levels could potentially correlate with both function and midline crossing. (4) Peripheral nerve stimulation has recently been shown to lead to subventricular zone (SVZ) neurogenesis (72), which has been implicated as a source of migrating neural progenitors to the lesioned motor cortex (73). Taken together, these possible mechanisms suggest that the neuroplastic effects promoted by peripheral nerve stimulation may not only act locally, but also likely involve multiple pathways and cellular mechanisms.

This study provides a reliable animal model to examine the effects of NMES on stroke recovery and to study the underlying mechanisms. There are several points worth mentioning for our model. (1) The nerve cuff electrode reduced animal discomfort during stimulation, allowed for a lower current amplitude (0.11 mA on average) to induce muscle contraction and targeted a specific nerve (median nerve in this study). (2) Utilization of a nerve cuff also allowed for the targeting of a specific nerve (median nerve in this study) and prevented current leakage into the adjacent nerves and tissues, thereby lessening activation of non-targeted nerves (74). (3) Our stimulation paradigm utilized a sub-tetany frequency (5 Hz) compared to other paradigms that utilize higher frequencies that may lead to muscle tetanus (75). Furthermore, a clinical study showed that low frequency stimulation led to activation of bilateral thalami (76) which connect to the sensorimotor cortex and may play an important role in neuroplastic changes. (4) We applied electrical stimulation for only 30 min each day and did not add any additional therapies, yet still yielded improvement in volitional function. Once it is translated for clinical use, the simplicity of this therapy promotes continued patient compliance. (5) Stimulation was delayed until 3 days after stroke, and therefore this intervention may be used in patients that are stabilizing post-stroke and/or missing the time window to receive tPA or thrombectomy. It is believed that most recovery from impairment in humans resulting from enhanced plasticity occurs in the first 1–3 months after stroke (25, 77). The cortical plastic responses to stroke are particularly active within days or weeks after stroke in rodents (78–80). Our reports showed that such plasticity could be enhanced when interventions were given ranging from within a week (18, 19, 32, 52, 53) to months post-stroke in rodents (20, 21). Therefore, the time window to deliver NMES to promote neuronal plasticity and improve functional outcome may be extended beyond the acute stage after stroke in both rodent models and human patients. However, as it is not known for certain, more studies are needed to determine the best time window for clinical use.

In conclusion, median nerve stimulation post-ischemic stroke led to functional recovery on sensorimotor tasks that can potentially be attributed to plastic changes at the cervical spinal cord level. This study has provided an important model for both the cellular mechanisms by which peripheral E-stim promotes functional recovery, and its stimulation paradigm which has high translation potential to the stroke population at large.

## Data Availability Statement

The raw data supporting the conclusions of this article will be made available by the authors, without undue reservation.

## Ethics Statement

The animal study was reviewed and approved by Hines VA/Lovell IACUC.

## Author Contributions

S-YT contributed to the concept and design, acquisition/analysis and interpretation of data, and drafted the major part of the article. JS contributed to acquisition/analysis and interpretation of data, and drafted part of the article. NA, JWu, and ST contributed to the acquisition/analysis of data. RH contributed to the design and experimental method refinement. JWa contributed to the design and refinement of experimental methods. TO'B contributed to data analysis/interpretation. GK contributed to the concept and design, interpretation of data, and revised the article for important intellectual content. RN originated the concept and design, interpretation of data, revised the article for important intellectual content. All the authors read and approved the final version.

## Conflict of Interest

The authors declare that the research was conducted in the absence of any commercial or financial relationships that could be construed as a potential conflict of interest.
